# Local Functional Connectivity as a Pre-Surgical Tool for Seizure Focus Identification in Non-Lesion, Focal Epilepsy

**DOI:** 10.3389/fneur.2013.00043

**Published:** 2013-05-01

**Authors:** K. E. Weaver, W. A. Chaovalitwongse, E. J. Novotny, A. Poliakov, T. G. Grabowski, J. G. Ojemann

**Affiliations:** ^1^Department of Radiology, University of WashingtonSeattle, WA, USA; ^2^Integrated Brain Imaging Center, University of WashingtonSeattle, WA, USA; ^3^Industrial and Systems Engineering, University of WashingtonSeattle, WA, USA; ^4^Neurology, Seattle Children’s HospitalSeattle, WA, USA; ^5^Radiology, Seattle Children’s HospitalSeattle, WA, USA; ^6^Department of Neurology, University of WashingtonSeattle, WA, USA; ^7^Department of Neurological Surgery, University of WashingtonSeattle, WA, USA; ^8^Neurosurgery, Seattle Children’s HospitalSeattle, WA, USA

**Keywords:** resting state fMRI, functional connectivity, non-lesion, focal epilepsy, ReHo, contralateral, pre-operative evaluation, epilepsy surgery

## Abstract

Successful resection of cortical tissue engendering seizure activity is efficacious for the treatment of refractory, focal epilepsy. The pre-operative localization of the seizure focus is therefore critical to yielding positive, post-operative outcomes. In a small proportion of focal epilepsy patients presenting with normal MRI, identification of the seizure focus is significantly more challenging. We examined the capacity of resting state functional MRI (rsfMRI) to identify the seizure focus in a group of four non-lesion, focal (NLF) epilepsy individuals. We predicted that computing patterns of local functional connectivity in and around the epileptogenic zone combined with a specific reference to the corresponding region within the contralateral hemisphere would reliably predict the location of the seizure focus. We first averaged voxel-wise regional homogeneity (ReHo) across regions of interest (ROIs) from a standardized, probabilistic atlas for each NLF subject as well as 16 age- and gender-matched controls. To examine contralateral effects, we computed a ratio of the mean pair-wise correlations of all voxels within a ROI with the corresponding contralateral region (IntraRegional Connectivity – IRC). For each subject, ROIs were ranked (from lowest to highest) on ReHo, IRC, and the mean of the two values. At the group level, we observed a significant decrease in the rank for ROI harboring the seizure focus for the ReHo rankings as well as for the mean rank. At the individual level, the seizure focus ReHo rank was within bottom 10% lowest ranked ROIs for all four NLF epilepsy patients and three out of the four for the IRC rankings. However, when the two ranks were combined (averaging across ReHo and IRC ranks and scalars), the seizure focus ROI was either the lowest or second lowest ranked ROI for three out of the four epilepsy subjects. This suggests that rsfMRI may serve as an adjunct pre-surgical tool, facilitating the identification of the seizure focus in focal epilepsy.

## Introduction

Current standards of care for the treatment of pharmacoresistant, focal epilepsy includes the surgical resection of epileptogenic cortex. Typically, the tissue targeted for resection encompasses an extended area around the seizure focus believed to be involved in the propagation of epileptiform discharges, generally referred to as the epileptogenic zone (Rosenow and Lüders, 2001; Laufs, 2012). The benefits of epilepsy surgery have clearly been established. Numerous prospective as well as longitudinal studies have shown that higher rates of seizure freedom, improved quality of life, and decreased long-term remission rates are associated with successful surgical intervention (Wiebe et al., 2001; Spencer and Huh, 2008; de Tisi et al., 2011).

The precise localization of the seizure focus and the extended epileptogenic zone is therefore critical to yielding positive, post-operative outcomes. Pre-surgical evaluations aimed at identifying the seizure focus are comprised of any number of interdisciplinary approaches, including electrophysiological investigations [e.g., electroencephalography and less frequently sub-dural electrophysiology such as electrocorticography (ECoG) or stereoelectroencephalography], traditional neuropsychological evaluation, modern structural [e.g., structural magnetic resonance imaging (MRI)], metabolic [e.g., [^18^F]fluoro-2-deoxy-glucose positron emission tomography (FDG-PET)], and functional imaging based approaches (e.g., functional MRI). The typical clinical evaluation identifies sites of pathology from structural-based MR scans and probes surrounding tissue for epileptogenic potential using a combination of the aforementioned modalities. However, in a few patients (i.e., approximately 25% of all qualifying surgical candidates), structural imaging is normal (i.e., an absence of qualitative, gross pathology – Duncan, 2010). In these non-lesional cases, seizure localization presents an additional challenge and clinicians must rely more heavily on alternative approaches (Siegel et al., 2001; Jayakar et al., 2008).

[18F]fluoro-2-deoxy-glucose positron emission tomography, which has traditionally been a widely used pre-surgical evaluative tool, plays a particularly important role in the absence of identified structural abnormalities (Mauguière and Ryvlin, 2004). In cases of refractory, non-lesional epilepsy, identification of a focal area of hypometabolism may reflect candidate seizure focus sites. It is not uncommon however to find hypometabolic regions outside the suspected region of interest (ROI). Thus, FDG-PET hypometabolic regions are frequently used to guide ECoG recordings. During these studies it is often noted that the extent of abnormal hypometabolic regions overlaps with the ictal onset zones and in many cases these areas are substantially larger than and overlap with electrodes displaying interictal epileptic discharges (IEDs) (Duncan, 2010). Moreover, overlapping sites of hypometabolism are commonly lateralized to one hemisphere. For example, when classified by seizure-freedom rates at a 12-month follow-up, quantitative comparisons of FDG uptake rates of the hypometabolic regions relative to the contralateral side showed high accuracy (∼80%) in identifying the hemisphere harboring the epileptogenic focus (Won et al., 1999).

In recent clinical research studies, fMRI has been shown to be a reliable complementary study to FDG-PET. For example, resected cortex displaying pre-operative evoked BOLD signal activations highly concordant with simultaneously EEG recorded IEDs was associated with a greater probability of post-operative seizure freedom (Thornton et al., 2010). It was noted that the greater the degree of overlap between resected tissue and the spread of IED correlated BOLD signal across a region, the greater the probability of long-term seizure freedom. Based on this good concordance, the authors suggested that use of simultaneously acquired EEG-fMRI maybe “a useful adjunct” during the pre-operative evaluation of epileptogenic cortex, particularly in the absence of identified pathology (Zijlmans et al., 2007; Thornton et al., 2010). Despite the major advantages of simultaneous EEG-fMRI during pre-operative evaluation, it is not readily available in the clinical setting.

One promising application of BOLD fMRI that may aid seizure focus localization and is now commonly available in the clinical setting is resting state functional MRI (rsfMRI) functional connectivity (fc) (Fox and Raichle, 2007; Biswal et al., 2010). This method calculates whole-brain voxel-wise correlations of infra-slow (<0.1 Hz) BOLD signal fluctuations extracted during a resting period and depicts them as maps of brain connectivity. rsfMRI has been used extensively to reveal patterns of fc across and between large-scale neural networks (Damoiseaux et al., 2006). These patterns of correlations are believed to reflect an underlying dynamic but intrinsic neural architecture (Honey et al., 2009; Keller et al., 2011) driven by direct (e.g., mono-synaptic) and/or indirect (poly-synaptic) anatomical connectivity (Biswal et al., 2010). Many proposed applications have capitalized on the inherent advantages of rsfMRI. For instance, rsfMRI has been to shown to identify intact language networks in the absence of verbal responses (Shimony et al., 2009). In MTLE patients, rsfMRI has revealed disrupted fc across regions commonly involved in the greater epilepsy network, primarily on the ipsilateral side to the seizure focus. Interestingly, increased fc within contralateral regions was also observed suggestive a possible cross-hemisphere compensatory mechanism (Bettus et al., 2009). As a follow-up investigation, the same group of investigators reported that fc increases observed contralateral to MTL pathology lead to high degree of specificity (>91%) for identification of the hemisphere that houses the seizure focus (Bettus et al., 2010).

More recent developments in rsfMRI methodology have begun to focus on patterns of connectivity specific to the local cortical environment (Zang et al., 2004). That is, measures of local connectivity mapping correlations restricted to a finite set of voxels within a ROI. One such method that has recently gained some popularity is Regional Homogeneity (ReHo), a technique that calculates a non-parametric cross-correlation coefficient between the time-series of a center voxel with a local cluster of voxels of pre-defined sized (Zang et al., 2004; Zhong et al., 2011). To date, reports applying ReHo for seizure focus localization have not been published. A few studies have contrasted ReHo in epilepsy patients relative to control volunteers, observing for example significantly higher thalamic ReHo in a group of generalized tonic-clonic epilepsy patients, values that were negatively correlated with epilepsy duration (Zhong et al., 2011). The anatomical assumptions underlying local fc are built upon patterns of cortico-cortical connectivity. Variability across local cortical neighborhoods or “small-world networks” are therefore assumed to reflect weighted differences of connectivity across neighboring neuronal units (He et al., 2007; Bullmore and Sporns, 2009) leading to the concept of scale-free network properties inherent to the brain’s innate architecture (Barabási and Albert, 1999).

While epileptogenic mechanisms and the underlying etiologies are widely variable in patients with focal, treatment-resistant epilepsy, it is well established that the aberrant nature of prolonged epileptic discharges lead to significant neuroanatomical alterations particularly within the epileptogenic zone (Thom, 2004). Animal models and neuropathological reports of resected human epileptogenic tissue have revealed that prolonged seizure activity results in (among many other well-established biochemical and pathological effects) significant neuronal injury and necrosis within the seizure network, particularly within neocortical pyramidal cells (Sankar et al., 1998; Chen and Wasterlain, 2006). Further, a wealth of animal studies has concluded that persistent seizures activity can lead to significant dendritic damage including alterations in spin morphology and an overall down regulation of dendritic spines (Multani et al., 1994; Wong and Guo, in press).

Our overall aim is to examine the capacity of rsfMRI local connectivity to serve as a useful adjunct in the pre-operative evaluation process of seizure focus localization. Based on the extent literature, we hypothesized that local fc in and around the seizure focus in patients with non-lesion, focal (NLF) epilepsy would be significantly lower relative to (1) controls, (2) the corresponding region within the contralateral hemisphere, and (3) ipsilateral ROIs outside of the epileptogenic zone. We choose a two-step analysis approach. First we examined fc in and around the seizure focus. To accomplish this we calculated whole-brain ReHo and averaged across different ROIs. We then tailored a more traditional fc approach to specifically contrast local fc at the seizure focus to the corresponding region within the contralateral hemisphere, an analysis we referred to as IntraRegional Connectivity (IRC).

## Materials and Methods

### Subjects

Four NLF epilepsy individuals (two female, mean age: 37.75 – Table 1) with unknown pathology (MRI negative) were scanned prior to epilepsy surgery at the University of Washington (UW). Scans from NLF patients were acquired on two different scanners (three on a clinical and one on a research magnet; both Philips 3T Achieva) using identical eight-channel SENSE head coils. Table 1 details the biographical information for each NLF subject. Note: color-coding within Table 1 is kept consistent throughout to denote results specific to each individual NLF subject. In order to minimize variance within the NLF data sets due to use of different scanners, we downloaded functional and anatomical data sets from 16 age- and gender-matched controls (Table A1 in Appendix) from a multisite rsfMRI repository, the 1000 connectomes database^1^. Of the 16 controls, one quarter were specifically matched to one NLF subject. That is, four gender-matched controls with an age range of ±1 year were selected with specific reference to each NLF subject.

**Table 1:** **Epilepsy subject demographic information and scanning parameters**.

	Subject	Age	Gender	Focus location	TR/TE	Resolution	No. of volumes	Matrix	No. of slices	Scanner
Epilepsy 1	fpei	34	F	Right inferior sub-temporal	2, 21	3.5 × 3.5	180	64 × 64	38	Philips 3T (clinical)
Epilepsy 2	FPE2	36	F	Right posterior sub-temporal	2, 21	3.5 × 3.5	180	64 × 64	38	Philips 3T (clinical)
Epilepsy 3	FPE3	37	M	Left medial to inferior temporal	2, 21	3 × 3	180	80 × 80	41	Philips 3T (research)
Epilepsy 4	FPE4	44	M	Left middle temporal	2, 21	3.5 × 3.5	180	64 × 64	38	Philips 3T (clinical)

### Imaging

#### MRI acquisition

At each UW scan session (NLF subjects), the scanning protocol included a Magnetization prepared rapid gradient echo (MPRAGE) high-resolution T1 sequence (repetition time (TR)/echo time (TE)/flip angle: 6.5 ms/3 ms/8°; matrix size of 256 × 256 and with 170 sagittally collected slices and a slice thickness of 1 mm) and a 6-min resting state, echo planar fMRI sequence (rsfMRI, TR/TE/FA: 2000/21/90°). The clinical scan sequence consisted of 38 axially oriented slices and a matrix size 64 × 64, while the research scan sequence consisted of 41 axially oriented slices and a matrix size 80 × 80. For all subjects, five “dummy” volumes which were collected to stabilize T1 equilibration effects were excluded from analyses. Scan parameters for the 1000 connectomes control subjects varied according to acquisition site (see Table A1 in Appendix for details).

#### Seizure focus identification

**Figure 1:**
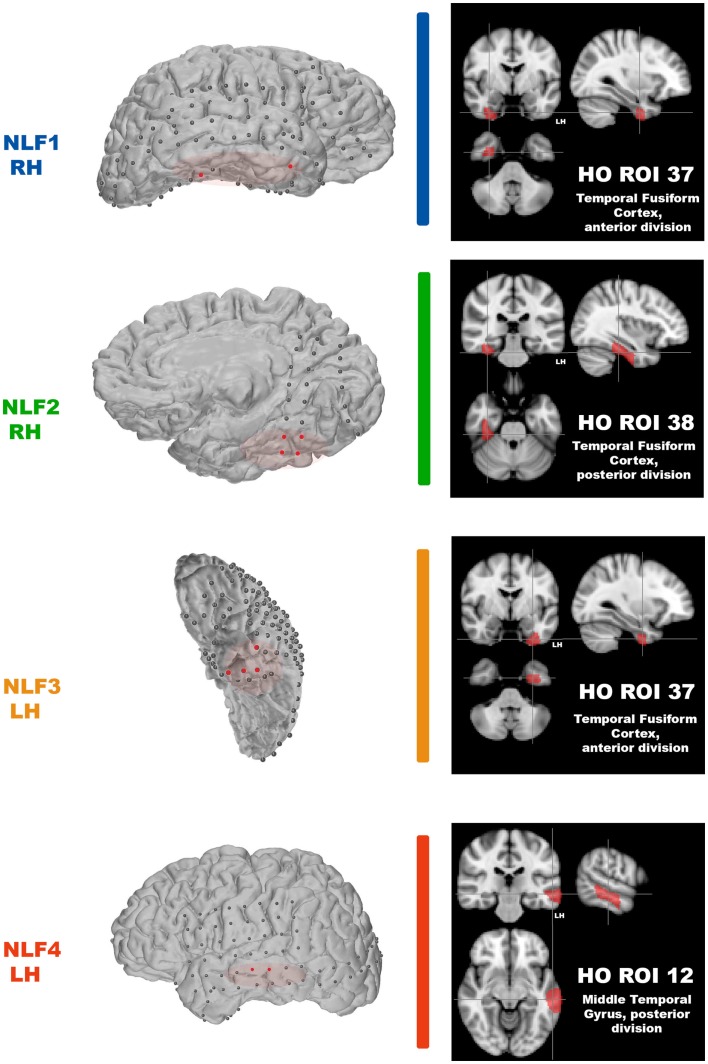
**Identification of the seizure focus and ROI**. For each NLF subject, the seizure focus was retrospectively defined and identified as tissue showing onset of ictal discharges based on electrocorticographic findings as well as contained with the resection zone (epileptogenic zone). For consistency throughout, each NLF subject is color-coded as seen in Table 1. First column shows a 3D surface rendering of the subjects high-res T1 MPRAGE scan (generated with FREESURFER automated tools for surface reconstruction) with the overlaid ECoG grid and strip electrodes. Electrodes colored in red reveal the locales of the ECoG recorded ictal onset activity. The overlaid red transparencies show the approximate resected, epileptogenic zone. The second column (black boxes) plots the HO ROI (on the MNI 152 brain) that overlaps with the electrode falling within the seizure focus for each NLF subject.

After scanning, each NLF epilepsy subject underwent a craniotomy and long-term ECoG monitoring for epileptiform discharges. Ictal onset was defined clinically from video-ECoG and identification of concordant fast spiking, low voltage activity extending from the sub-dural montage. Figure 1 (left column) shows the ECoG montage for the four NLF subjects. Electrodes highlighted in red denote the electrodes in the ictal onset zone. After ECoG monitoring, subjects underwent surgical resection of epileptic tissue. The red transparent areas (Figure 1, left column) reveal the approximate location of the resected tissue as outlined by post-op surgical notes. The location of the seizure focus was defined as the region containing an overlap between ECoG recorded ictal onset activity contained within the resection zone.

### Analysis

#### Pre-processing

At the individual level, standard rsfMRI pre-processing was conducted using FEAT (FMRI Expert Analysis Tool) Version 5.98, part of FSL (FMRIB’s Software Library)^2^ to remove non-neuronal sources of variance. These included skull stripping using BET, motion correction (realignment to the center volume) with FSL MCFLIRT, spatial smoothing using a 6 mm full-width half-maximum (FWHM) Gaussian kernel, grand-mean intensity normalization, and linear drift removal. Identified volumes exceeding 0.5 mm of motion in any direction or plane were eliminated (scrubbed) from further processing. Additionally, ventricular CSF signal was extracted, averaged, and removed from the overall whole-brain time-series. Each 4D data set was entered into a regression analysis, treating the movement parameters and CSF signal as nuisance variables. Finally, to limit the effect of physiological noise on fc, the overall time-series was temporally low-passed filtered removing frequencies above 0.1 Hz.

#### Regions of interests

**Figure 2:**
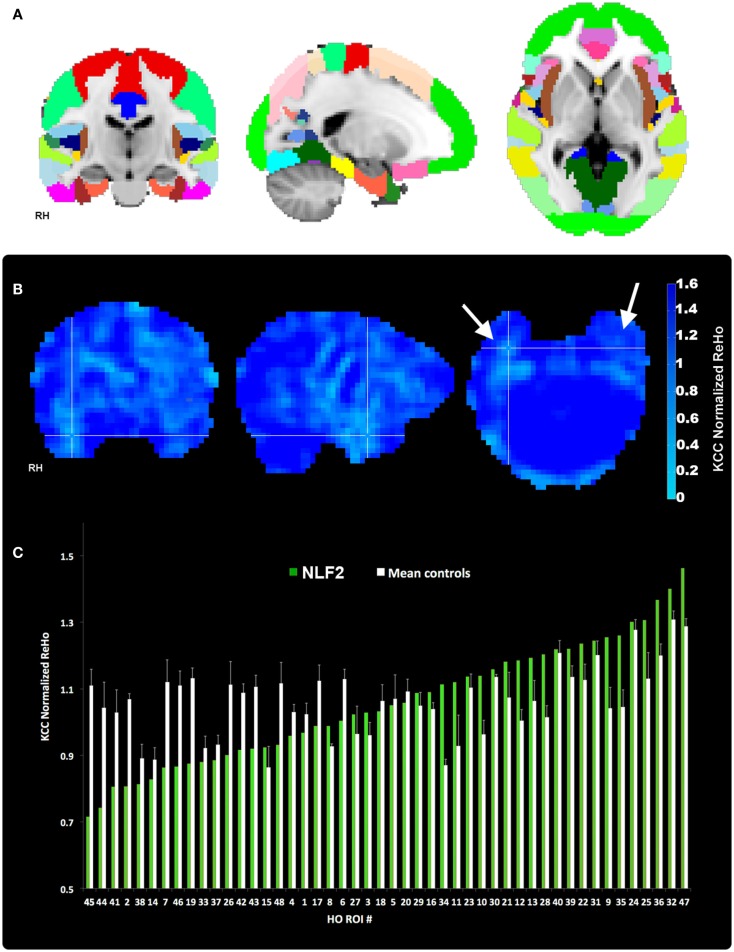
**Regions of interest and local connectivity**. **(A)** Shows the 48 thresholded HO ROIs overlaid in the MNI 152 brain. Using the structural detail inherent to the high-res T1 scans, all ROIs were warped into native fMRI space for each subject. Whole brain, normalized ReHo, and IRC values were then extracted and averaged from each ROI. **(B)** Reveals an example ReHo map form one epilepsy subject. Note the cross-hairs pinpoint a qualitative decrease in ReHo in and around the seizure focus within the right hemisphere, an effect that is absent from the left. **(C)** Plots raw normalized ReHo values across the 48 HO ROIs for the same NLF subject (green bars) and the mean of the four age- and gender-matched control subjects (white bars). Epilepsy and control values are sorted from lowest to highest for the NLF subject. In this NLF subject, the ROI that contains the epileptogenic zone (ROI 38) has one of the five lowest mean normalized ReHo values of all ROIs.

Our aim was to compare across cortical regions containing the seizure focus and control regions at the individual level. Thus, we parcellated each individual subject’s brain into established, known ROIs using the MNI Harvard–Oxford (HO) probability atlas (included as part of the FSL anatomical toolkit; Figure 2A). Each of the 48 HO cortical ROIs (employing the 25% threshold criteria) were selected, degraded by an additional 25% to prevent overlap after warping into native space and then co-registered into native fMRI space through a three-step registration process using FSL FLIRT. First, the native high-resolution MPRAGE was registered into native fMRI space using a rigid-body transform. Second, the MNI 2-mm standard brain was registered onto the warped MRPRAGE using an affine transformation. The generated transformation matrices from standard-to-warped MPRAGE were then applied to all HO ROIs. Finally, for each patient the HO co-registered ROI that contained the electrode overlaying the seizure focus was identified and selected for statistical analysis (Figure 2, right-most column, cross-hairs, the ROI corresponding to the seizure focus is listed in the bottom right hand corner of the black box).

### FC analyses

#### Regional homogeneity

Each 4D pre-processed data set was then passed through ReHo analysis using the REST toolbox in MATLAB^3^. For each voxel, a mean correlation coefficient was computed using Kendal’s coefficient of concordance (KCC-ReHo), relative to the time-series from the surrounding 27 voxel neighbors. Voxel-wise ReHo values were normalized by dividing by the global mean KCC-ReHo value (Mankinen et al., 2011). Greater ReHo values denote increased local connectivity (Figure 2B).

#### IRC

To specifically contrast fc between the HO ROI contralateral to the seizure focus, we adapted traditional fc methods by computing pair-wise correlation coefficients between all possible voxel pairs within each HO ROI. Coefficients within an ROI were then transformed into *z*-scores and a mean value of absolute *z*-scores was estimated. This score was then transformed back into an average correlation coefficient yielding a mean value of intra-nodal or local fc. Finally, a ratio of local fc in the left hemisphere ROI to the connectivity in the right hemisphere ROI was calculated. If the ratio is close to 1, the brain’s fc is more symmetric, vice versa. The ratios were subsequently converted into log scale resulting in degrees of asymmetry (i.e., the larger the value, the more left ROI is locally connected in comparison to the right ROI).

#### Statistics

We are specifically interested in whether local fc within the HO ROI containing the seizure focus is lower relative to the same ROI in controls and non-seizure focus ROIs within each epilepsy subject (thus serving as his/her own control). Because of the small patient population presenting with refractory, non-lesion epilepsy combined with interest in comparing across different fc analyses, we used a non-parametric ranking metric to evaluate differences at the group level. For each subject, HO ROIs were ranked from lowest to highest with respect to the normalized ReHo values ipsilateral to the hemisphere housing the seizure focus (for an example ranking see Figure 2C). IRC ROIs were sorted according to the degree of left-to-right (or right-to-left depending on which hemisphere housed the focus) asymmetry. The two rankings were then averaged. Thus, stemming from our local connectivity analysis approaches, we generated three sets of rankings of 48 values of local connectivity for each subject. The rank value of each focus ROI for each of the three rankings were entered into an independent sample Wilcoxon Rank Sum test (two-sided, alpha level of 0.05), contrasting the rank value of that ROI for the four NLF against the 16 matched controls.

Additionally, we reasoned the translational value of rsfMRI fc as a pre-operative evaluation tool would come at the individual level, contrasting local fc values across brain regions for a given surgical candidate. To characterize the ranking values for each NLF epilepsy subject, we took a parametric approach calculating the mean and standard deviation of the ranks from across all controls for each of the four seizure focus ROIs. For each of these four distributions, a corresponding *z*-score and *p* value was estimated testing the null hypothesis that the local fc rank for a given NLF epilepsy subject was no different than the controls rank values.

Finally, the mean ReHo values from each ROI was standardized to a −1 to 1 distribution in order to average across the quantitative estimate of ReHo with the left-right IRC ratios (Table 2). For each HO ROI, a mean standardized ReHo and IRC ratios were averaged, ranked, and compared to the mean rank values.

**Table 2:** **ReHo, IRC and mean ranking values for all HO ROIs**.

ReHo												IRC							
1			2			3			4				1		2		3		4	
ROI	Raw value	Normalized	ROI	Raw value	Normalized	ROI	Raw value	Normalized	ROI	Raw value	Normalized		ROI	IRC	ROI	IRC	ROI	IRC	ROI	IRC
**NLF SUBJECT**																				
34	0.658	1.000	45	0.716	1.000	***37***	0.794	1.000	14	0.735	1.000		34	1.000	2	1.000	25	1.000	6	1.000
***37***	0.668	0.974	44	0.742	0.929	34	0.819	0.905	11	0.761	0.932		7	0.856	46	0.924	6	0.925	41	0.937
14	0.710	0.851	41	0.806	0.759	27	0.854	0.770	5	0.821	0.772		***37***	0.615	***38***	0.823	***37***	0.714	42	0.911
41	0.732	0.789	2	0.807	0.755	8	0.913	0.541	9	0.848	0.700		14	0.300	48	0.507	48	0.624	27	0.838
38	0.738	0.771	***38***	0.814	0.736	14	0.926	0.491	***12***	0.896	0.575		6	0.239	1	0.462	28	0.508	4	0.802
27	0.771	0.676	14	0.827	0.701	38	0.939	0.441	33	0.896	0.575		42	0.236	41	0.424	5	0.500	25	0.774
42	0.800	0.593	7	0.863	0.605	25	0.943	0.425	15	0.909	0.541		1	0.155	21	0.390	26	0.455	40	0.772
2	0.808	0.570	46	0.867	0.596	3	0.952	0.391	38	0.910	0.539		27	0.134	19	0.386	36	0.447	9	0.764
35	0.854	0.439	19	0.875	0.574	1	0.954	0.384	34	0.923	0.503		38	0.097	43	0.363	40	0.408	19	0.756
46	0.854	0.438	33	0.880	0.560	33	0.975	0.303	8	0.931	0.481		18	−0.036	8	0.335	7	0.391	22	0.731
8	0.865	0.406	37	0.886	0.544	29	0.979	0.289	37	0.950	0.432		19	−0.055	42	0.308	27	0.384	2	0.727
33	0.874	0.379	26	0.902	0.503	28	1.002	0.198	44	0.971	0.377		20	−0.075	33	0.285	19	0.355	24	0.716
43	0.887	0.343	42	0.916	0.463	26	1.006	0.183	26	0.972	0.373		29	−0.099	4	0.270	39	0.355	47	0.700
7	0.890	0.335	43	0.920	0.454	35	1.011	0.162	27	0.973	0.370		17	−0.125	34	0.179	13	0.348	32	0.695
15	0.898	0.310	15	0.924	0443	4	1.025	0.108	35	0.988	0.332		16	−0.129	7	0.135	24	0.338	28	0.678
29	0.906	0.287	48	0.932	0.422	15	1.048	0.021	45	0.994	0.315		10	−0.149	14	0.131	30	0.325	31	0.676
44	0.919	0.252	4	0.959	0.350	11	1.050	0.012	1	1.002	0.295		2	−0.155	40	0.123	23	0.323	39	0.632
45	0.923	0.239	1	0.968	0.326	6	1.064	−0.039	10	1.020	0.248		21	−0.175	44	0.095	32	0.309	29	0.624
6	0.928	0.224	17	0.989	0.269	7	1.067	−0.052	4	1.026	0.232		46	−0.193	26	0.089	46	0.298	45	0.622
5	0.938	0.195	8	0.989	0.269	5	1.070	−0.064	3	1.037	0.203		23	−0.205	10	0.066	34	0.297	20	0.615
1	0.970	0.103	6	1.004	0.227	41	1.079	−0.098	20	1.044	0.184		39	−0.222	16	0.037	10	0.281	17	0.581
10	0.979	0.079	27	1.023	0.176	32	1.086	−0.126	46	1.047	0.176		8	−0.247	31	0.000	43	0.249	3	0.574
18	0.990	0.047	3	1.029	0.163	36	1.097	−0.169	19	1.047	0.176		44	−0.288	20	−0.002	31	0.244	44	0.564
17	0.999	0.020	18	1.032	0.154	10	1.105	−0.200	6	1.050	0.167		3	−0.294	39	−0.025	17	0.238	23	0.536
28	1.005	0.002	5	1.051	0.103	30	1.106	−0.202	41	1.059	0.146		33	−0.320	30	−0.056	20	0.227	33	0.515
4	1.025	−0.055	20	1.058	0.085	18	1.109	−0.216	2	1.067	0.125		31	−0.372	47	−0.059	3	0.221	18	0.508
32	1.034	−0.079	29	1.088	0.004	31	1.117	−0.246	18	1.084	0.078		5	−0.402	12	−0.060	21	0.217	36	0.506
19	1.048	−0.122	16	1.090	−0.002	47	1.120	−0.257	42	1.085	0.076		35	−0.409	23	−0.082	29	0.185	35	0.477
11	1.060	−0.156	34	1.114	−0.065	9	1.122	−0.265	36	1.087	0.072		15	−0.413	36	−0.104	15	0.113	30	0.477
3	1.063	−0.163	11	1.120	−0.083	16	1.128	−0.287	30	1.089	0.066		48	−0.452	32	−0.115	47	0.110	46	0.415
26	1.069	−0.180	23	1.136	−0.126	17	1.143	−0.345	7	1.093	0.055		32	−0.460	3	−0.126	44	0.048	43	0.400
21	1.073	−0.192	10	1.139	−0.133	46	1.150	−0.372	48	1.099	0.038		36	−0.478	25	−0.129	1	0.045	15	0.376
20	1.083	−0.222	30	1.159	−0.186	39	1.162	−0.419	43	1.100	0.035		12	−0.519	45	−0.158	35	0.034	37	0.375
48	1.084	−0.224	21	1.182	−0.248	24	1.165	−0.431	29	1.107	0.018		24	−0.522	37	−0.158	45	0.013	26	0.367
47	1.100	−0.271	12	1.186	−0.259	42	1.170	−0.448	28	1.111	0.007		45	−0.535	11	−0.160	4	0.010	21	0.358
16	1.101	−0.273	13	1.193	−0.277	40	1.171	−0.454	17	1.116	−0.006		28	−0.544	24	−0.210	9	−0.018	34	0.345
12	1.113	−0.308	28	1.204	−0.305	2	1.172	−0.457	32	1.146	−0.085		43	−0.575	18	−0.249	41	−0.043	16	0.328
30	1.130	−0.357	40	1.220	−0.349	48	1.179	−0.484	23	1.148	−0.091		30	−0.625	17	−0.249	42	−0.058	48	0.312
9	1.132	−0.361	39	1.220	−0.351	12	1.184	−0.502	40	1.151	−0.098		*22*	−0.626	29	−0.269	14	−0.070	10	0.305
39	1.139	−0.382	22	1.236	−0.393	13	1.192	−0.535	13	1.154	−0.107		26	−0.655	15	−0.279	I8	−0.133	1	0.235
23	1.139	−0.383	31	1.245	−0.415	44	1.202	−0.574	16	1.183	−0.182		4	−0.669	27	−0.306	33	−0.209	***12***	0.233
24	1.152	−0.420	9	1.255	−0.444	19	1.208	−0.597	39	1.183	−0.183		13	−0.678	13	−0.389	38	−0.211	14	0.125
31	1.187	−0.519	35	1.260	−0.457	45	1.212	−0.612	21	1.229	−0.303		40	−0.698	*22*	−0.401	12	−0.218	5	0.035
13	1.196	−0.545	24	1.302	−0.570	22	1.228	−0.673	24	1.235	−0.319		47	−0.709	5	−0.536	2	−0.225	38	−0.017
36	1.219	−0.612	25	1.307	−0.582	23	1.229	−0.676	22	1.281	−0442		25	−0.717	35	−0.613	16	−0.282	11	−0.036
***22***	1.255	−0.715	36	1.367	−0.744	43	1.262	−0.804	31	1.336	−0.586		9	−0.864	6	−0.635	22	−0.328	7	−0.076
25	1.288	−0.811	32	1.401	−0.834	20	1.292	−0.921	47	1.388	−0.724		41	−0.879	28	−0.640	8	−0.989	13	−0.088
40	1.354	−1.000	47	1.463	−1.000	21	1.313	−1.000	25	1.493	−1.000		11	−1.000	9	−1.000	11	−1.000	8	−1.000
1			2			3			4		
ROI	Mean rank	Mean value	ROI	Mean rank	Mean value	ROI	Mean rank	Mean value	ROI	Mean rank	Mean value
**NLF SUBJECT**											
34	1	1.000	2	2.5	0.877	***37***	2	0.857	9	6	0.732
***37***	2.5	0.794	***38***	4	0.780	25	4	0.712	27	9	0.604
7	3.5	0.595	46	4.5	0.760	34	7	0.601	6	12	0.584
14	6.5	0.575	41	5	0.592	77	8.5	0.577	14	12.5	0.562
38	7	0.434	44	8.5	0.512	6	10	0.443	33	13.5	0.545
42	7	0.415	19	10	0.480	78	10	0.353	41	15.5	0.542
27	8	0.405	48	10	0.464	26	11	0.319	4	15.5	0.517
6	12	0.232	33	11	0.422	3	13	0.306	42	16	0.493
2	12.5	0.207	45	11	0.421	29	14.5	0.237	44	17.5	0.471
1	14	0.129	14	11	0.416	5	15.5	0.218	45	17.5	0.469
46	14.5	0.122	43	11.5	0.408	1	17	0.215	19	18.5	0.466
29	14.5	0.094	1	11.5	0.394	14	19.5	0.210	15	19.5	0.459
8	16.5	0.080	42	12	0.386	7	20	0.170	11	20.5	0.448
33	16.5	0.029	7	15	0.370	36	20.5	0.139	2	21	0.426
35	18.5	0.015	4	15	0.310	38	20.5	0.115	34	21.5	0.424
18	18.5	0.005	8	15.5	0.302	35	21	0.098	35	21.5	0.405
44	19	−0.018	26	17	0.296	32	22	0.092	***12***	22	0.404
10	19	−0.035	37	20.5	0.193	48	22.5	0.070	37	22.5	0.404
41	19.5	−0.045	15	21.5	0.082	15	22.5	0.067	5	23	0.403
15	20	−0.052	21	22.5	0.071	30	22.5	0.062	70	23	0.399
17	22	−0.052	34	24.5	0.057	4	23	0.059	3	23	0.388
19	22.5	−0.088	20	24.5	0.042	33	23.5	0.047	26	23.5	0.370
5	23.5	−0.103	3	26	0.019	10	24	0.041	28	23.5	0.342
43	25	−0.116	16	27	0.017	31	24.5	−0.001	40	25	0.337
45	25	−0.148	17	27.5	0.010	40	25	−0.023	29	25.5	0.321
20	25.5	−0.148	10	27.5	−0.033	39	25	−0.032	32	26	0.305
21	25.5	−0.184	18	28.5	−0.048	46	25.5	−0.037	46	26	0.296
16	26.5	−0.201	27	29	−0.065	74	25.5	−0.047	18	26	0.293
3	27	−0.229	23	29.5	−0.104	17	25.5	−0.053	36	26.5	0.289
32	29	−0.270	40	30.5	−0.113	41	27	−0.071	17	27	0.287
28	30.5	−0.271	11	31	−0.121	47	27	−0.073	10	27.5	0.276
23	30.5	−0.294	30	31.5	−0.121	13	27.5	−0.093	30	28	0.271
39	30.5	−0.302	29	31.5	−0.132	19	29	−0.121	1	28	0.265
48	32	−0.338	12	31.5	−0.160	9	29	−0.142	38	28.5	0.261
4	33.5	−0.362	39	32.5	−0.188	18	31	−0.174	39	28.5	0.224
12	34.5	−0.414	6	33	−0.204	23	32.5	−0.177	23	28.5	0.223
26	35	43.417	31	33.5	−0.208	8	32.5	−0.224	43	29	0.218
31	35.5	−0.446	5	34.5	−0.217	42	33	−0.253	24	29.5	0.199
24	38	−0.471	13	37	−0.333	44	34	−0.263	48	29.5	0.175
47	38	−0.490	25	37.5	−0.356	43	36	−0.277	22	30	0.144
30	38.5	−0.491	24	38.5	−0.390	16	36	−0.284	16	31	0.073
36	38.5	−0.545	77	38.5	−0.397	45	36.5	−0.300	31	31	0.045
11	39.5	−0.578	36	39	−0.424	2	37.5	−0.341	21	32	0.027
13	42.5	−0.612	28	40	−0.473	20	37.5	−0.347	7	35	−0.011
9	42.5	−0.613	32	41.5	−0.475	12	38.5	−0.360	47	38.5	−0.012
22	43	−0.671	47	42	−0.530	21	40.5	−0.392	13	39	−0.097
25	45.5	−0.764	35	44	−0.535	11	41	−0.494	25	39	−0.113
40	46	−0.849	9	45	−0.722	22	45	−0.501	8	43.5	−0.259

*Raw values for local fc measurements with the corresponding rankings are shown across all ROIs. Red, bold text represents the seizure focus ROI for each subject*.

## Results

**Figure 3:**
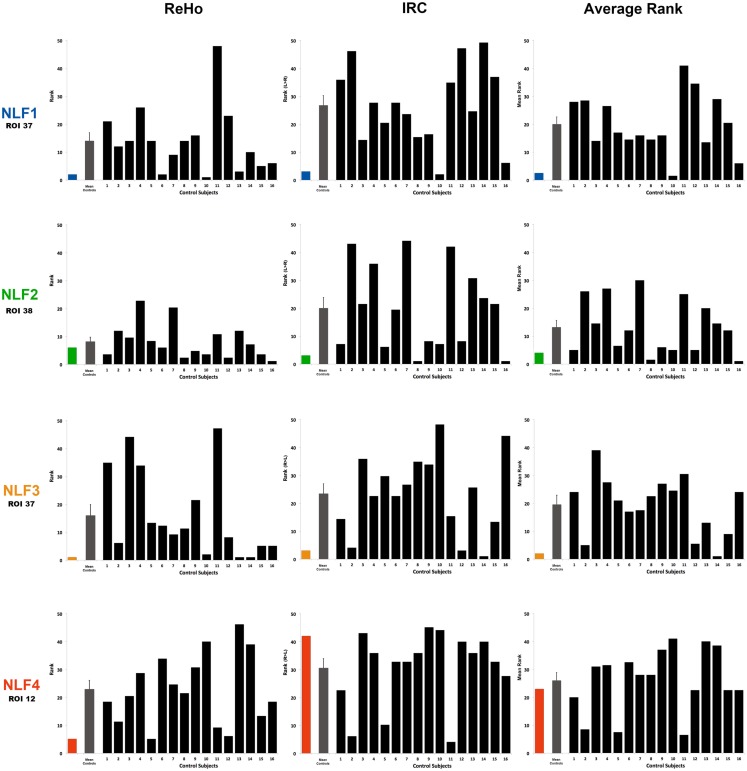
**Ranking the local fc estimates from the HO ROI around the seizure focus**. For each NLF subject and the 16 age- and gender-matched controls, ReHo, IRC, and mean scalars were calculated from the ROI that contained the seizure focus. For each of the 20 subjects, values from all ROIs were sorted from lowest to highest and assigned a rank relative to the 48 ROIs within the HO atlas. The first column plots ReHo ranks, the second column plots the IRC ranks (ranking either R > L or L > R) and the third the average rank across the two methods for all subjects. The color bar represents the ranking for the respective NLF subject as noted in Table 1. The gray bar represents the mean (with standard error of the mean) of the 16 controls subjects, and each black bar represents the ranking for each control subject.

The HO ROI containing the seizure focus for each epilepsy subject ordered in the bottom 10% for all within-subject rankings except for the IRC ranking for NLF4 (red text in Table 2). For example, the ReHo ranking for participant NLF1 was 2 indicating the HO ROI housing the seizure focus had the second lowest mean, normalized ReHo with respect to all ipsilateral ROIs. Further, the IRC ranking for this subject was 3, indicating that this ROI showed the third lowest local fc ranking when mean local fc was directly contrasted with its contralateral counterpart. The one exception was the IRC ranking for subject NLF4, indicated that the local fc showed a greater degree of contralateral connectivity relative to the seizure focus. Figure 3 plots the rank value for the three ranking distributions revealing the raw values for each non-lesional, focal epilepsy patient as the colored bar.

### Group-level contrasts

To determine whether local fc in the seizure focus ROI was lower in the epilepsy group, we compared the rank value of the seizure focus ROI between NLF and controls across our three sets of rankings (ReHo, IRC, and mean rank). Both the ReHo (*p* = 0.0156, Wilcoxon Rank Sum test) and the mean rank (*p* = 0.0421, Wilcoxon Rank Sum test) were significantly lower averaged across the NLF subjects (Figure 3, color bars) relative to controls (Figure 3, mean value shown in gray bars) but not the IRC fc method (*p* = 0.0184). It should be noted that the unusual contralateral connectivity effect seen with in NLF4 subject likely contributed to the null statistical effect for the IRC method at the group level.

### Individual-level contrasts

To piece out ranking effects at the individual level, we calculated *z*-score statistics from the mean and SD across ranks values from the controls. For each seizure focus ROI across each of the three local fc rankings, we were able to reject the null hypothesis for only NLF1 subject (*p* = 0.0424) under the IRC rankings. Further, when the ReHo and IRC rankings were averaged together, both subjects NLF1 (*p* = 0.0409) and NLF3 (*p* = 0.0427) showed significantly lower rankings relative to controls.

We also directly contrasted the mean rankings (i.e., the average between ReHo and IRC) for each individual NLF subject with a mean value of the raw local fc estimations. For each ROI, quantitative local fc values were an average metric calculated from the normalized ReHo and the IRC ratio scores. The mean local fc value paralleled the average ranking for all four NLF epilepsy subjects. The red text items in Table 2 reveal the ranking and raw local fc values for each of the seizure focus ROIs. As can be seen, across both the mean rankings and combined local fc estimates, the ROI housing the seizure focus was lower in value relative to either of the constituent values alone for three out of the four NLF patients. For example, in the patient NLF 2, the seizure focus ROI was the second lowest ranked and second lowest combined computed local fc value, but ranked third and fifth when individually sorting IRC and ReHo values, respectively.

## Discussion

Here we observe that rsfMRI local fc shows some potential as a pre-operative mapping tool for seizure focus identification in individuals with NLF epilepsy. We examined two different methods of fc estimation, averaged across both methods and contrasted local fc at the site of the seizure focus between epilepsy individuals, normal controls, and within-subject ROIs. At a group level, we observed a decrease in both the ReHo ranking and the combined rank for the ROI harboring the focus compared to a matched group of control subjects. This suggests that in our cohort of local epileptics there was a marked decrease in one measure of local fc (ReHo) in the area around the seizure focus. Thus, at the group level, the disease process associated with epilepsy appears to alter local fc around the focus, a hypothesis that is consistent with the pathological effects typically seen in the epileptogenic zone (Thom, 2004; Wong and Guo, in press). The real clinical value however of local fc to epilepsy surgery is an accurate estimation of the location of the epileptogenic zone at the individual level that is concordant with other modalities of investigation. This is particularly important for patients with focal epilepsy with a normal MRI where macroscopic structural abnormalities are not available as an initial guide for surgical planning.

### A within-subject method for identifying the seizure focus

We therefore examined whether rsfMRI could clarify the location of the seizure focus in NLF epilepsy at an individual level. Based on neuropathological reports and animal studies of focal epilepsy reporting significant neuronal necrosis at the seizure focus, we hypothesized that values of local connectivity would be abnormal in and around the seizure focus (Thom, 2004; Wong and Guo, in press). Based upon an extensive imaging literature showing compensatory effects within the contralateral hemisphere, we extended this hypothesis to a specific decrease in local fc within the ipsilateral relative to the contralateral cortical region (Won et al., 1999; Morgan et al., 2012). The approach providing the greatest potential for revealing our predicted effects was averaging across both ReHo and IRC and contrasting across all ROIs from a single subject (Table 2). This procedure revealed that for three out of the four NLF subjects the seizure focus ROI was either the lowest (NLF3) or second lowest (NLF1 and NLF2) ranked ROI (see Figure 3, colored bars, Table 2; for a specific discussion on the IRC ranking of NLF4, see below). That is, the predictive capacity of local fc rsfMRI in focal, non-lesion epilepsy is improved when combining a method that specifically computes local fc within the region around the seizure focus (ReHo) with an analysis that contrasts local fc with specific reference to the corresponding contralateral hemisphere (IRC).

We argue using the epilepsy patient as his or her own control while combining these two local fc approaches provides the most promise as a translational tool. rsfMRI provides whole-brain coverage. Thus, contrasting ReHo and IRC values across the brain is readily available when employing standard clinical rsfMRI sequences. Normative, population values for patterns of local fc have not been established, more importantly are not readily available in the clinical setting and will likely need to be developed for specific MR systems and imaging sequences. When combined with established physiological and anatomical and functional imaging abnormalities commonly associated with the seizure focus as well as including the possible compensatory effects observed in the contralateral hemisphere (Bettus et al., 2009), it is not surprising that factoring in both of these methodologies would improve the overall ability to identify the epileptogenic focus ROI.

This combined approach may also have an important role in patients early in the course of the epileptogenic process where potentially surgically remediable lesions can be identified at an incipient stage before neuroanatomical changes are observed on conventional MRI. Several studies have shown that surgical interventions early in the course of pharmacoresistant epilepsy leads to better quality of life and outcomes (Engel et al., 2012).

### The larger seizure network

For NLF1, the HO ROI 34 (corresponding to anterior division of the parahippocampal gyrus) ranked lower after combining both ReHo and the IRC methods than the seizure focus ROI (corresponding to the anterior extent of the temporal fusiform – Figure 1). Portions of HO ROI 34 were resected in this patient. Thus under established criteria, the parahippocampal gyrus would be included as part of the epileptogenic zone (c.f. Laufs, 2012). This region shares significant inter-connectivity with cortex throughout the medial temporal lobe, including the perirhinal and entorhinal cortices as well as with the hippocampus proper (Burwell, 2000). Accordingly, the parahippocampal cortex is heavily involved in recall and/or numerous memory-related processes (Eichenbaum et al., 2007). Intrinsic connectivity studies using rsfMRI have revealed significant fc with numerous neocortical association cortices including the posterior regions of the default mode network as well as inter-connectivity spread throughout the lateral temporal lobe (Ranganath and Ritchey, 2012) and extensively with the anterior extent of the inferior temporal lobe (Kahn et al., 2008). Not surprisingly, the parahippocampal gyrus is a key fixture in the larger network underlying MTL epilepsy and seizure propagation (McIntyre and Gilby, 2008). The widespread pattern of connectivity extending from the parahippocampal region throughout the temporal lobe provides an architecture that would easily promote temporal lobe seizure propagation. With specific reference to NLF1, the seizure focus is located in a densely connected adjacent portion of the anterior, inferior temporal lobe (the temporal lobe fusiform). Thus, the observation that these two regions show the lowest local fc estimates likely signifies that rsfMRI is revealing a broader epileptogenic zone or epilepsy network in this subject.

For NLF epilepsy subject 2, only the insular cortex ROI ranked lower in local fc relative to the seizure focus ROI (located within the posterior temporal fusiform). The insula is generally considered a multimodal integration site that shares a high level of connectivity with frontal and temporal cortex. A recent seed-based rsfMRI report noted significant fc between two different points along the anterior-posterior insular plane and the posterior fusiform (Taylor et al., 2009). Both ictal and IEDs originating from the insula have been reported in MTL epilepsy (Isnard et al., 2000). In this same report, it was observed that two patients with significant insular discharges continued to have seizures after temporal lobectomy. Moreover, lesions in the insula have been shown to develop into intractable epilepsy where resection of the lesion and the surrounding insular tissue yields seizure freedom (Roper et al., 1993). Based on these and similar reports, insula-based epilepsy has become more routinely recognized over the past few decades (Nguyen et al., 2009).

The converging notion from the current NLF epilepsy patients one through three is that alterations in local fc may identify the epileptogenic zone as well as the larger epilepsy network (Stufflebeam et al., 2011). The concept of widespread epilepsy networks has been identified using both imaging with MRS (Pan et al., 2012), SPECT (Sequeira et al., 2013), FDG-PET (Mauguière and Ryvlin, 2004), and electrophysiological studies (Muldoon et al., 2013). The observation that ROIs ranking lower in local fc relative to the seizure focus likely share rich patterns of connectivity with the seizure focus may be exposing a more widespread pathological consequence of the seizure propagation. Building upon the hypothesis that discrepancies in local fc are linked to local neuronal insults such as necrosis (or apoptosis), alterations in dendritic morphology, and potential compensation within the contralateral hemisphere, the currently applied techniques may be revealing the downstream consequences of seizure propagation across the entire epilepsy network.

### Methodological considerations and limitations

We choose to focus specifically on refractory, non-lesion epilepsy patients because of the added importance that functional-based modalities (i.e., electrophysiological and imaging based procedures) provide in the pre-surgical localization of the seizure focus. The number of patients presenting with NLF epilepsy that are candidates for surgery are however relatively small (<10% of all new cases per year; Duncan, 2010). Despite this limitation, the current results should be taken with a degree of caution due the small sample size. As a follow-up, future studies will clearly need to conduct similar analyses with larger samples. It is however likely that estimates of local fc may aid in the identification of the epileptogenic focus among patients presenting with various focal pathologies (i.e., cortical dysplasia, AVM, brain tumors etc.). Taken together with the lateralized fc differences throughout the medial temporal lobe previously reported in MTLE patients (Bettus et al., 2009), local fc would likely contribute to the pre-surgical evaluation even in the presence of an identified insult.

The current results would benefit from a more precise delineation of the epileptogenic zone. Other groups have identified the epileptogenic zone using a variety of additional techniques (c.f. Jayakar et al., 2008; Duncan, 2010). We were not able to use a more sophisticated means of defining the epileptogenic zone other than a description from post-op surgical notes of the extent and boundaries of the resected region. By choosing to parcellate the brain into ROIs using a well-established, probabilistic atlas combined with a sorting method based on mean local fc values, we ensured a completely unbiased process of identifying patterns of reduced local fc across subjects while maintaining relatively high anatomical specificity. One unfortunate and likely consequence of this procedure is a smearing of voxel types within an ROI. More specifically, it is unlikely that the ROI corresponding to the seizure focus in any given NLF patient contains voxels that would be exclusively labeled as falling in or exclusively out of the epileptogenic zone. Thus, it is likely that the mean values for each ROI in and around the epileptogenic zone are underestimated, and the true local fc value associated with the epileptogenic zone is likely lower. One possible solution for consideration in future studies is to contrast pre and post-resection MRI scans. This would generate a voxel mask of the resected tissue and by extension the extended epileptogenic zone. Furthermore, the current results would indeed benefit from the addition of simultaneously acquired EEG. Confirmation of the IED-related activity during rsfMRI acquisition would provide the ability to confirm the boundaries of epileptogenic zone. Provided the presence of IEDs during functional scanning, it may be feasible to select out specific periods of “IED-free” rsfMRI activity in order to determine whether the presence of IEDs are negatively (or positively) impacting local lc correlation coefficients. However, we reason that rsfMRI provides a simple yet powerful means of examining the underlying physiology of the epileptogenic zone that is also feasible in the clinical context (c.f. Fox and Greicius, 2010). Future studies will clearly need to address the influence of IEDs (as well as ictal discharges) on the rsfMRI BOLD activity and local fc estimates. Furthermore, future studies will need to address the concordance between rsfMRI local fc estimates in NLF epilepsy and more commonly used modalities such as FDG-PET. However, if local fc does indeed reflect the accurate location of the seizure focus and thereby supplementing more traditional evaluative modalities, then the need of simultaneous EEG would prove relatively superfluous.

NLF 4 did this not show the same pattern of IRC within the seizure focus ROI (located within the left middle temporal gyrus) as was observed in other NLF 3 patients. Although the raw and ranked ReHo values were within the bottom 10% of all sorted ROIs, the pattern of local fc under the IRC calculation was significantly greater within the ipsilateral hemisphere. The mechanism contributing to this effect is unknown. Results from the WADA test as well as clinical fMRI scans using various language screens concluded that language dominance was localized to the left hemisphere for this patient. It is possible that patterns of contralateral connectivity are not as vast within the middle, temporal lobe relative to noted contralateral compensatory effects stemming from medial temporal lobe (Bettus et al., 2009). It is also conceivable that scalars of local fc are greater in regions throughout the language dominant hemisphere relative to the contralateral counterparts. It is clear that future work will need to address baseline differences in local fc across both the temporal lobe as well as whole brain.

## Conclusion

We present evidence suggesting local fc measurements from rsfMRI provide an accurate estimate of the location of the epileptogenic region in non-lesional, focal epilepsy. Structurally identified lesions are typically considered a reliable guide as a first pass for identifying the approximate location of the epileptogenic zone. Because the long-term benefits of epilepsy surgery are significant for individuals presenting with normal anatomical MRIs (Jayakar et al., 2008), accurate localization is a critical pre-operative function. In the absence of identified lesions, clinicians must rely more heavily on alterative methods to identify epileptogenic zones. Here we provide the first evidence that rsfMRI local fc may provide additional, confirmatory information about the location of the epileptogenic focus in refractory NLF epilepsy. These techniques may also identify the broader epilepsy network and identify comorbid neuropsychological dysfunction due to involvement of other functional networks.

## Conflict of Interest Statement

The authors declare that the research was conducted in the absence of any commercial or financial relationships that could be construed as a potential conflict of interest.

## Footnotes

^1^http://fcon_1000.projects.nitrc.org

^2^www.fmrib.ox.ac.uk/fsl

^3^http://www.restfmri.net/forum/index.php

## Appendix

**Table A1:** **Control subject demographic information and scanning parameters**.

	1000C ID	Age	Gender	Control ID	TR	Resolution	No. of volumes	Matrix	No. of slices
NY	sub33062	34	F	ctrl002	2	3 × 3	180	64 × 64	39
Leipzig	sub41241	34	F	ctrl003	2.3	3 × 3	180	64 × 64	34
Palo Alto	sub29935	33	F	ctrl004	2	3.4 × 3.4	180	64 × 64	29
NY	sub53710	34	F	ctrl015	2	3 × 3	180	64 × 64	39
NY	sub30860	35	F	ctrl016	2	3 × 3	180	64 × 64	39
NY	sub47633	37	F	ctrl017	2	3 × 3	180	64 × 64	39
Oxford	subl3304	35	F	ctrl018	2	3 × 3	175	64 × 64	34
Oxford	sub85152	35	F	ctrl019	2	3 × 3	175	64 × 64	34
Bangor	sub04097	36	M	ctrl006	2	3 × 3	180	80 × 80	34
Bangor	sub81464	38	M	ctrl007	2	3 × 3	180	80 × 80	34
ICBM	sub51677	37	M	ctrl008	2	4 × 4	128	64 × 64	23
Leipzig	sub36858	38	M	ctrl009	2.3	3 × 3	180	64 × 64	34
ICBM	sub94169	43	M	ctrlOll	2	4 × 4	128	64 × 64	23
Milwaukie	sub91468	44	M	ctrl014	2	3.75 × 3.75	175	64 × 64	20
Milwaukie	sub49975	45	M	ctrl020	2	3.75 × 3.75	175	64 × 64	20
UW	sub56994	43	M	ctrl021	2	3 × 3	180	80 × 80	41
